# Genetic diversity and relationships of Chinese donkeys using microsatellite markers

**DOI:** 10.5194/aab-62-181-2019

**Published:** 2019-04-15

**Authors:** Lulan Zeng, Ruihua Dang, Hong Dong, Fangyu Li, Hong Chen, Chuzhao Lei

**Affiliations:** 1 College of Animal Science and Technology, Northwest A&F University, Yangling, Shaanxi, 712100, China; 2 College of Animal Science and Technology, Shihezi University, Shihezi, Xinjiang, 832003, China

## Abstract

Donkeys are one important livestock in China because of their nourishment and medical values. To investigate the genetic diversity and phylogenetic relationships of Chinese donkey breeds, a panel of 25 fluorescently labeled microsatellite markers was applied to genotype 504 animals from 12 Chinese donkey breeds. A total of 226 alleles were detected, and the expected heterozygosity ranged from 0.6315 (Guanzhong) to 0.6999 (Jiami). The mean value of the polymorphism information content, observed number of alleles, and expected number of alleles for all the tested Chinese donkeys were 0.6600, 6.890, and 3.700, respectively, suggesting that Chinese indigenous donkeys have relatively abundant genetic diversity. Although there were abundant genetic variations found, the genetic differentiation between the Chinese donkey breeds was relatively low, which displayed only 5.99 % of the total genetic variance among different breeds. The principal coordinates analysis clearly splits 12 donkey breeds into two major groups. The first group included Xiji, Xinjiang, Liangzhou, Kulun, and Guanzhong donkey breeds. In the other group, Gunsha, Dezhou, Biyang, Taihang, Jiami, Qingyang, and Qinghai donkeys were clustered together. This grouping pattern was further supported by structure analysis and neighbor-joining tree analysis. Furthermore, genetic relationships between different donkey breeds identified in this study were corresponded to their geographic distribution and breeding history. Our results provide comprehensive and precise baseline information for further research on preservation and utilization of Chinese domestic donkeys.

## 1 Introduction

Donkeys played an important role in ancient transport systems of Asia and Africa, donkeys provided a reliable source of protein and facilitated overland circulation of goods and people. China has a 4000-year history of raising donkeys (Zheng, 1985; Xie, 1987), and possesses more than 9 million donkeys, accounting for about 22 % of the world's donkey population (Hou and Hou, 2002). Twenty-four donkey breeds thrive throughout central, northeastern, and western China, primarily in the dry, arid, semi-arid, and warm climates of western China around the Yellow River valley, resulting in an abundant genetic resource (Xie, 1987). However, since the 1980s, the number of donkeys has been decreasing steadily along with agricultural mechanization. Moreover, some donkey breeds are currently threatened with extinction (Ma et al., 2003), such as the famous Guanzhong donkeys (Lei et al., 2007). Several studies have been conducted to investigate genetic diversity and origins of Chinese donkeys. Uniparental markers are routinely used to trace the origins of Chinese donkey breeds by defining paternal and maternal lineages on the basis of variation sites, which has revealed an African origin of Chinese donkeys (Chen et al., 2006; Han et al., 2014, 2017).

Autosomal microsatellite markers have been widely used in revealing genetic variability and identifying the genetic relationships among donkey populations (Jordana et al., 2001; Matassino et al., 2014; Rosenbom et al., 2015). Bordonaro et al. (2012) described the genetic variability and differentiation in Pantesco and two other Sicilian autochthonous donkey breeds by microsatellites makers. Recently, Jordana et al. (2016, 2017) analyzed genetic diversity and structure of American donkeys, providing information on putative routes of the spreading of donkeys across the American continent. These studies all provide important data for further breed-specific management and conservation programs.

In order to investigate the genetic diversity and population structure of Chinese indigenous donkeys, 504 animals from 12 native breeds were assessed using 25 fluorescently labeled microsatellite markers. The results present accurate and comprehensive insights into the genetic variation, genetic structure, and dispersal route of Chinese donkey breeds, contributing to a rational basis for working out breeding strategies and genetic conservation plans.

## 2 Methods

### 2.1 Sample collection and DNA extraction

**Figure 1:**
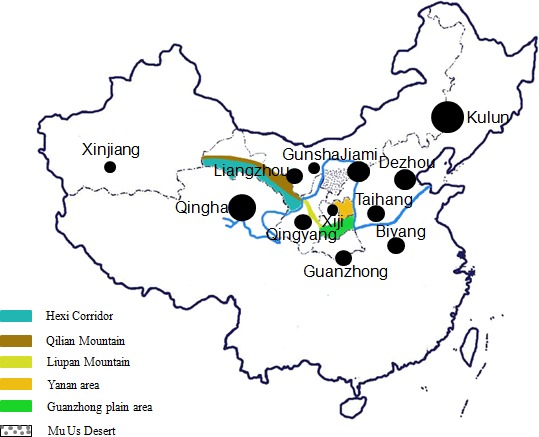
Geographical distribution of 12 Chinese donkey breeds.

A total of 504 individuals from 12 Chinese donkey breeds were collected, including two large donkey types (Dezhou, Guanzhong,), three medium types (Qingyang, Biyang, and Jiami), and seven small types (Kulun, Gunsha, Qinghai, Liangzhou, Xinjiang, Taihang, and Xiji). These breeds are distributed along the Yellow River basin and Guanzhong Plain (Fig. 1), which represent the major genetic resources of Chinese donkey breeds. Our aim was to collect at least 30 samples from a minimum of two separate flocks, although this was not possible for all breeds (more information about these breeds is showed in Table 1). The genomic DNA was isolated from peripheral blood using a standard phenol–chloroform protocol and stored at -20 ${}^{\circ }$C (Samhrook et al., 1989).

### 2.2 Microsatellite genotyping

A total of 25 microsatellite loci from previous studies were used: HTG7, HTG6, HMS7, HMS3, HMS2, AHT4, COR071, HMS6, UM011, NVHEQ18, HTG9, HTG15, ASB41, AHT21, LEX003, UCDEQ505, COR082, COR058, SGCV28, COR022, HMS45, ASB23, COR018, UCDEQ425, and ASB02 (Marklund et al., 1994; Røed et al., 1997; Locke et al., 2002; Ling et al., 2011) (Table S1 in the Supplement). Polymerase chain reaction (PCR) was performed in 12.5 µL reactions containing 10 ng template DNA, 20 pM of each primer, 6.5 µL 2× PCR mix buffer (0.75 U Taq DNA polymerase, 2× PCR buffer, 37.5 µM ${\mathrm{MgCl}}_{2}$, and 5 µM dNTPs) (PE Applied Biosystems, MA, USA). The PCR cycles were initial denaturation at 95 ${}^{\circ }$C for 5 min; 30 cycles of denaturation at 94 ${}^{\circ }$C for 30 s, primer annealing at the optimal temperature for each primer pair (ranged from 55 to 68 ${}^{\circ }$C, Table S1) for 30 s, and extension at 72 ${}^{\circ }$C for 30 s; final extension at 72 ${}^{\circ }$C for 10 min and stored at 4 ${}^{\circ }$C. After PCR amplification, amplified fragments were separated by capillary electrophoresis using an ABI PRISM 3100 automatic sequencer (Applied Biosystems, Foster City, CA, USA) and allele sizing was accomplished by using the internal size standard GeneScan^™^-500LIZ^™^. Fluorescently labeled fragments were detected and sized using Peak Scanner v1.0.2.3.

### 2.3 Statistical analysis

A Fisher's exact test was performed to determine possible deviation from the Hardy–Weinberg equilibrium (HWE) using GENEPOP 1.2 (Raymond and Rousset, 1995). Exact p values were estimated from the Markov-chain algorithm using 10 000 dememorization steps, 500 batches, and 5000 iterations per batch. Population genetic indexes, such as the observed number of alleles ($N_{a}$), effective number of alleles ($N_{e}$), observed heterozygosity ($H_{o}$), and expected heterozygosity ($H_{e}$) of each donkey breed, were obtained using POPGENE 1.31 software (Yeh et al., 1999). The F-statistic values ($F_{IS}$, fixation indices of subpopulation; $F_{IT}$, fixation indices of total population; $F_{ST}$, fixation index resulting from comparing subpopulations to the total population; Weir and Cockerham, 1984), together with the total number of alleles (At), were estimated with Arlequin version 3.1 (http://cmpg.unibe.ch/software/arlequin3, last access: 27 February 2019). The polymorphic information content (PIC) of each locus was calculated using PIC CALC (Nagy et al., 2012). The number of private alleles (NPA) was counted using the GDA program (https://download.csdn.net/download/vip8_8/9856774, last access: 27 February 2019)

A principal coordinates analysis (PCoA) was performed to reveal major patterns of genetic variability and clustering of breeds based on $F_{ST}$ matrix using GENALEX 6.501 (Peakall and Smouse, 2006). The population structure of the Chinese donkey was investigated by STRUCTURE (http://web.stanford.edu/group/pritchardlab/structure.html, last access: 4 March 2019). Each run included a burn-in period of 800 000 Markov chain Monte Carlo (MCMC) steps, followed by 1 000 000 additional iteration steps. Neighbor-joining (NJ) trees were constructed based on the weighted estimator of Reynolds' distance (DR; Reynolds et al., 1983) by using POPULATIONS version 1.2.30 (Langella, 2002). The robustness of the dendrograms was evaluated using a bootstrap test of 5000 resembling of loci, with replacement. The unrooted distance tree was then visualized with TREEVIEW version 1.6.6 (Page, 1996).

## 3 Results

### 3.1 Polymorphism of microsatellite loci

All of the microsatellite loci were amplified and were polymorphic in 12 donkey breeds. The HWE was tested for all breed–locus combinations, significant (P<0.05) deviations from a HWE were observed for 158 (13.50 %) of 300 breed–locus combinations (Table S3). On average, 13.16 alleles per breed and 4.080 breeds per locus deviated significantly from HWE. The Gunsha and Qinghai donkeys showed the maximum number of loci in disequilibrium (19 loci), followed by Qingyang donkey (17 loci).

Of the 25 microsatellite loci analyzed, as many as 262 alleles were identified for the studied donkey populations (Table S2). The total number of alleles per locus (AT) ranged from 3 (HTG6 and COR022) to 20 (AHT4), with a mean of 10.48. PIC is an index of gene abundance, the level of which indicates the diversity of the genetic basis of a breed. PIC reflects genetic variation in microsatellite loci. When PIC >0.5, 0.5> PIC >0.25, and PIC <0.25, it indicates the locus has high polymorphism, moderate polymorphic, and low polymorphism, respectively (Botstein et al., 1980). The PIC across the 25 loci ranged between 0.1489 (COR022) and 0.8670 (HMS2). Additionally, 20 loci showed high polymorphism (PIC >0.5) and three loci (SGCV28, HMS45, and ASB02) showed moderate polymorphism (PIC >0.25) (Table S2).

### 3.2 Genetic diversity among native Chinese donkey breeds

A summary of the identified polymorphisms from 12 donkey breeds is listed in Table 1. Various alleles in a population are attributed to the long-term evolution. The mean $N_{a}$ for 12 Chinese donkey breeds was 6.890, ranging from 5.720 (Gunsha) to 8.120 (Kulun). The $N_{e}$ was the highest in the Jiami breed (4.320) and lowest in the Guanzhong breed (3.280), with a mean of 3.700. Heterozygosity (H), also known as genetic diversity, reflects the genetic variation on N loci, which is generally considered to be the optimal parameter for estimating genetic variation in a population. $H_{o}$ for the whole population was 0.5708 that showed a range of values from 0.5397 (Qingyang) to 0.5993 (Kulun). The $H_{e}$ values varied between 0.6315 in Guanzhong donkeys and 0.6999 in Jiami donkeys (mean value =0.6628), which showed no significant difference among breeds (Table 1).

A total of 32 private alleles were observed in our study (Table 1); the NPA of the Qinghai donkey was particularly high (NPA =9), representing 28.12 % of the total NPA. However, half of the donkey breeds have only one private allele that was at very low frequencies of below 4 % and no private alleles were detected in Guanzhong and Gunsha donkeys. The inbreeding coefficients ($F_{IS}$) of all Chinese donkey breeds were positive, and the values of five Chinese breeds (Dezhou, Liangzhou, Jiami, Qinhai, and Qingyang; $F_{IS}>0.0750$) differed significantly from zero (P<0.01). These results indicate the possibility of inbreeding within the population, evoking the necessity to carefully select a proper strategy for further conservation of the resource.

### 3.3 Genetic distance and relationship among native Chinese donkey breeds

**Figure 2:**
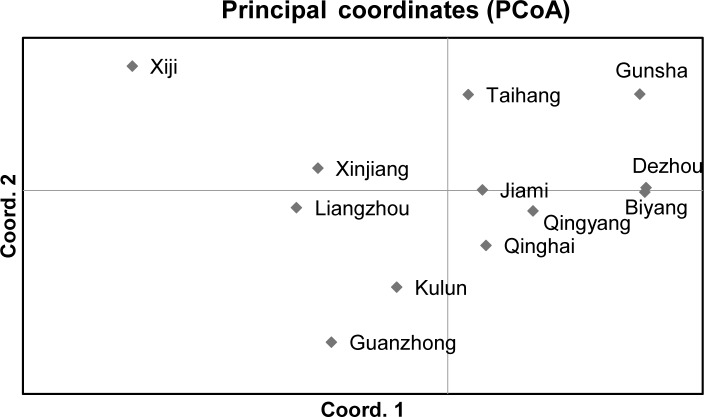
Principal coordinates analysis (PCoA) of 12 Chinese indigenous donkey breeds.

The PCoA method was performed to investigate possible genetic relationships between Chinese donkey breeds (Fig. 2). The first axis (accounting for 27.88 % of variation) separated two groups. The first group encompassed Xiji, Xinjiang, Liangzhou, Kulun, and Guanzhong donkeys. The second one gathered Gunsha, Dezhou, Biyang, Taihang, Jiami, Qingyang, and Qinghai donkeys. The second axis (19.54 %) tended to separate the Xiji donkey breed from the other donkeys of the first group.

**Figure 3:**
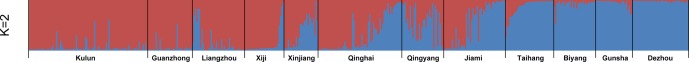
Population structures of 12 Chinese indigenous donkey breeds assuming K=2.

The results of the STRUCTURE program analysis revealed that there were two geographical lineages when K=2 (Fig. 3). The existence of two major clusters was consistent with the PCoA analysis, such that the first inferred one (cluster A) gathered Kulun, Guanzhong, Liangzhou, and Xiji donkey breeds, the second one (cluster B) included Biyang, Dezhou, and Gunsha donkeys, while other donkey breeds (Qingyang, Qinghai, Jiami, Xinjiang, and Taihang) had contributions from both clusters. According to the results with K=4 (Table S5), the Xiji population seems to have evolved independently due to inefficient transportation, and has experienced a genetic drift process.

**Figure 4:**
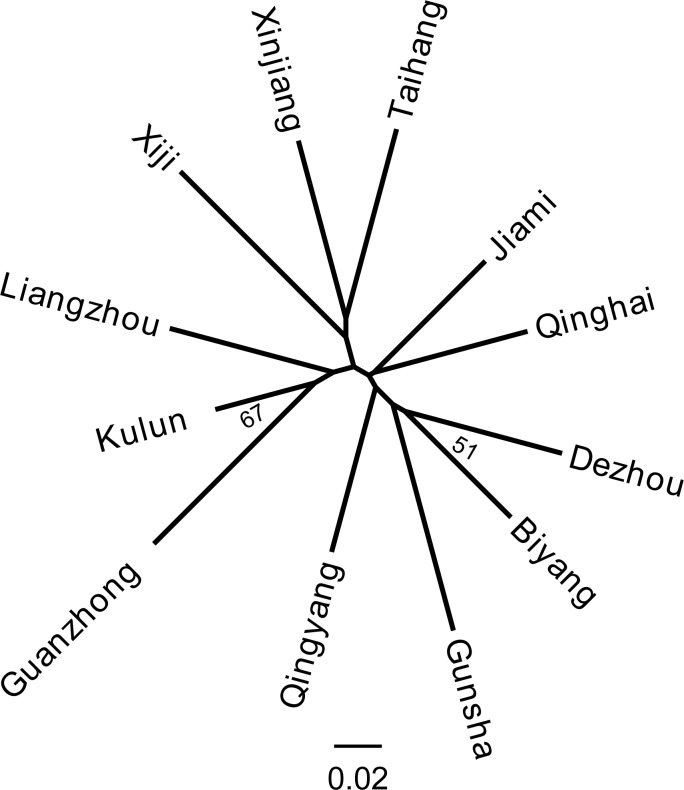
Neighbor-joining tree based on DR of 12 Chinese donkey breeds.

Genetic distance is a measure of genetic variation between populations, which objectively reflects variations and differentiation between them. An NJ tree was constructed on the basis of the Reynolds' distance. It showed that all 12 donkey breeds could be clustered into two clusters (Fig. 4), which highly correspond to the results of PCoA and structure analysis (K=2).

## 4 Discussion

### 4.1 Genetic diversity and differentiation of Chinese donkeys

In this study, the polymorphisms at 25 microsatellite loci in 504 Chinese donkeys from 12 breeds were investigated. The overall and average $N_{a}$ were very high, reflecting relatively high genetic variability in these donkey breeds. Among Chinese donkeys, the $H_{e}$ ranged from 0.6315 (Guanzhong) to 0.6999 (Jiami), which showed a comparable level to the previous values reported in Spanish (Arangurenméndez et al., 2001) and Croatian coast donkeys (Ivankovic et al., 2015), and was more diversified than Poitou (Bellone et al., 2002), Italian (Colli et al., 2013; Matassino et al., 2014) and American donkeys (Jordana et al., 2016).

There was a wide range of values concerning NPA among Chinese donkey breeds. The Qinghai donkey had particularly high NPA values. Furthermore, there were eight Chinese donkey breeds that had less than two private alleles. Additionally, the results of F statistics in the donkey populations showed that over half of the breed–locus combinations deviated from HWE (P<0.05; Table S3). This might be due to a predominance of mating between close relatives or small effective population sizes in these donkey breeds. With the enhancement of agricultural mechanization during the last four decades, the Chinese donkey population suffered from a severe reduction in population size (Ma et al., 2003). As a result, available breeding males were limited.

Genetic differentiation among the breeds was characterized by estimating overall and pairwise $F_{ST}$ values. The total $F_{ST}$ of Chinese donkey breeds is 0.0599, suggesting that 94.11 % of the total genetic variation resulted from genetic differentiation within breeds (Table 1), which showed a higher value compared to Italian donkeys (Colli et al., 2013; Matassino et al., 2014), but lower than that of donkeys in Africa (Rosenbom et al., 2015) and America (Jordana et al., 2016). Our results indicated a moderate degree of population differentiation in Chinese donkey breeds.

**Table 1:** Basic parameters for the genetic diversity associated with 12 donkey breeds.

Type	Breeds	Sample	Locality	NPA	$N_{a}$	$N_{e}$	$H_{o}$	$H_{e}$	$F_{IS}$
		size							
large	Dezhou	43	Dezhou City, Shandong Province	5	6.56	3.64	0.5622	0.6484	0.0899${}^{c}$
	Guanzhong	34	Fufeng County, Shaanxi Province	0	6.04	3.28	0.5734	0.6315	0.0317
	Qingyang	32	Qingyang City, Gansu Province	1	7.16	4.05	0.5397	0.6789	0.1467${}^{c}$
medium	Biyang	32	Biyang County, Henan Province	1	6.52	3.66	0.5942	0.6453	0.0371
	Jiami	47	Mizhi County, Shaanxi Province	7	7.68	4.32	0.5651	0.6999	0.1402${}^{c}$
	Liangzhou	40	Wuwei City, Gansu Province	1	7.28	3.83	0.5618	0.6908	0.1298${}^{c}$
	Gunsha	28	Yulin City, Shaanxi Province	0	5.72	3.52	0.5687	0.6492	0.0394
	Kulun	91	Chifeng City, Inner Mongolia region	5	8.12	3.69	0.5993	0.6614	0.0406${}^{b}$
small	Qinghai	64	Gonghe County, Qinghai Province	9	8.00	3.89	0.5564	0.6781	0.0758${}^{c}$
	Taihang	37	Linzhou City, Henan Province	1	6.92	3.34	0.5817	0.6429	0.0519${}^{a}$
	Xiji	30	Xiji County, Ningxia region	1	6.40	3.60	0.5921	0.6568	0.0602${}^{a}$
	Xinjiang	26	Yining City, Xinjiang region	1	6.24	3.60	0.5551	0.6701	0.1279${}^{c}$
	Mean	504		32	6.89	3.70	0.5708	0.6628	

n is number of population; NPA is number of private alleles; $N_{a}$ is observed number of alleles; $N_{e}$ is effective number of alleles; $H_{o}$ is observed heterozygosity; $H_{e}$ is expected heterozygosity; $F_{IS}$ is population inbreeding coefficient. ${}^{a}$ P<0.05; ${}^{b}$ P<0.01; ${}^{c}$ P<0.001.

### 4.2 Relationship among 12 Chinese native donkey breeds

In this study, the analysis with the STRUCTURE program revealed that Chinese donkeys were grouped into two lineages when K=2 (Fig. 3): cluster A included Kulun, Guanzhong, Liangzhou, and Xiji donkey breeds and cluster B gathered Dezhou, Gunsha, Biyang, and Taihang breeds, while other donkey breeds (Xinjiang, Qinghai, Qingyang, and Jiami) appeared to be the contact zone between both clusters, as individuals had mixed lineages. The results support the previous genetic research about the origin of the Chinese donkey, in which Chinese donkeys have two distinct mitochondrial maternal lineages, known as Nubian wild ass (*Equus africanus africanus*) and the Somali wild ass (*Equus africanus somaliensis*) (Lei et al., 2007; Han et al., 2014). When K=3 (Fig. S1), Taihang donkeys were separated within cluster B and have a genetic relationship with Xinjiang donkeys, which is presumably the result of an ancient founder effect that took place at the early stages of colonization. In addition, the joint influence of isolation and selection pressure may also contribute to particular phenotypes. According to the results of structure analysis (K=4; Fig. S1), the Xiji population seems to have evolved independently due to inefficient transportation and has experienced a genetic drift process. Indeed, the Xiji breed is a unique genetic resource with nearly 100 years breeding history. They are today still bred in Xiji County of the Ningxia Hui Autonomous Region with complex landforms and limited traffic conditions. Furthermore, Xiji donkeys are mainly breeding in restricted and small populations by local people. The government introduced the Guanzhong donkey in 1964, but the influence was low. After that, Xiji donkeys never crossed with any other donkey breeds (China National Commission of Animal Genetic Resources, 2011). All of these reasons may contribute to Xiji donkeys differing from other 11 Chinese donkey breeds.

The NJ tree and PCoA also recapitulated these findings that all 12 donkey breeds could be clustered into two groups (Figs. 4 and 2). Additionally, two main groups suggest that the colonization process and expansion of donkeys across China followed at least two main pathways. According to textual research and ancient DNA studies (Han et al., 2014), the earliest domestic Chinese donkeys were from the small donkeys of ancient Xinjiang and entered the mainland 2000 years ago (west Han Dynasty). They arrived in the Hexi Corridor of the northern Qilian Mountains along the Silk Road and then developed into Liangzhou donkeys. After entering the west of Liupan mountain, they lived in Xiji County of the Ningxia Hui Autonomous Region and its environs. They adapted to the semi-arid mountainous climate and developed into the Xiji donkey (Yang, 1991).

Based on the historical record, the Silk Road of the Song Dynasty (1000 years ago) entered the central plains and was not from the Hexi Corridor but from the Yan'an area (close to the Guanzhong Plain area). Therefore, donkeys of western regions could adapt well to the alpine steppe ecological types in the specific ecological environment of the Mu Us Desert and developed into Kulun donkeys, which might contribute to the close relationship between Guanzhong and Kulun donkey breeds (Fig. 3). The results of the NJ tree showed that Xinjiang, Liangzhou, Xiji, Guanzhong, Kulun, and Taihang donkeys are clustered together, which is consistent with their geographical distribution and breeding history.

During the Tang Dynasty, when the Silk Road reached its golden age, the number of Chinese domestic donkeys had increased primarily to meet the demand for the expansion of trade (Han et al., 2014). After arriving in the Guanzhong Plain area (the Chang'an, now Xi'an city was the center of politics, economy and culture in ancient China), donkeys of the western regions were rapidly imported to Qinghai, Shaanxi, Henan, Hebei, and Shandong provinces along the Yellow River Basin, and developed into the famous Qinghai, Jiami, Gunsha, Biyang, Qingyang, and Dezhou donkey breeds (Yang and Hong, 1989). Therefore, these donkey breeds clustered into another group (Fig. 4)

Our results also support the previous hypothesis for three dispersal routes of Chinese donkeys: (1) the spread of Chinese domestic donkeys in history was from Xinjiang via Ningxia, Gansu to the Guanzhong Plain of Shaanxi Province; (2) at the same time, Chinese domestic donkeys dispersed in parallel from Xinjiang to Inner Mongolian and Yunnan Province; (3) finally, Chinese domestic donkeys dispersed from Guanzhong Plain to other regions of China (Lei et al., 2007).

## 5 Conclusions

To conclude, these results reveal an insight into the genetic diversity and relationships between the Chinese donkeys, which demonstrated that indigenous donkey populations of China retain relatively abundant genetic diversity and the genetic relationships between different donkey breeds correspond to their geographic distribution and breeding history. The information presented here will be used to optimize reproductive management and provide tools for adopting adequate breeding strategies aimed at preserving its genetic variability.

## Supplement

10.5194/aab-62-181-2019-supplement The supplement related to this article is available online at: https://doi.org/10.5194/aab-62-181-2019-supplement.

## Data Availability

The data sets are available upon request from the corresponding author.
