# Supplementary material for: Genetic diversity and relationships of Chinese donkeys using microsatellite markers
Source: Arch Anim Breed. 2019 Apr 15;62(1):181–7. doi: 10.5194/aab-62-181-2019 (PMC6853031; doi:10.5194/aab-62-181-2019)
Supplement: The supplement related to this article is available online at: https://doi.org/10.5194/aab-62-181-2019-supplement. [file aab-62-181-supplement.zip › aab-62-181-2019-supplement-title-page.pdf]

Supplement of Arch. Anim. Breed., 62, 181–187, 2019
https://doi.org/10.5194/aab-62-181-2019-supplement
© Author(s) 2019. This work is distributed under
the Creative Commons Attribution 4.0 License.

Open Access
Archives Animal Breeding

*Supplement of*

# Genetic diversity and relationships of Chinese donkeys using microsatellite markers

**Lulan Zeng et al.**

*Correspondence to:* Chuzhao Lei (leichuzhao1118@126.com)

- aab-62-181-2019-supplement-title-page.pdf
- Supplement
  - Fig S1.tif
  - Supplemental Files.xlsx

The copyright of individual parts of the supplement might differ from the CC BY 4.0 License.
