# Supplementary figures and images for: Genetic diversity and relationships of Chinese donkeys using microsatellite markers
Source: Arch Anim Breed. 2019 Apr 15;62(1):181–7. doi: 10.5194/aab-62-181-2019 (PMC6853031; doi:10.5194/aab-62-181-2019)

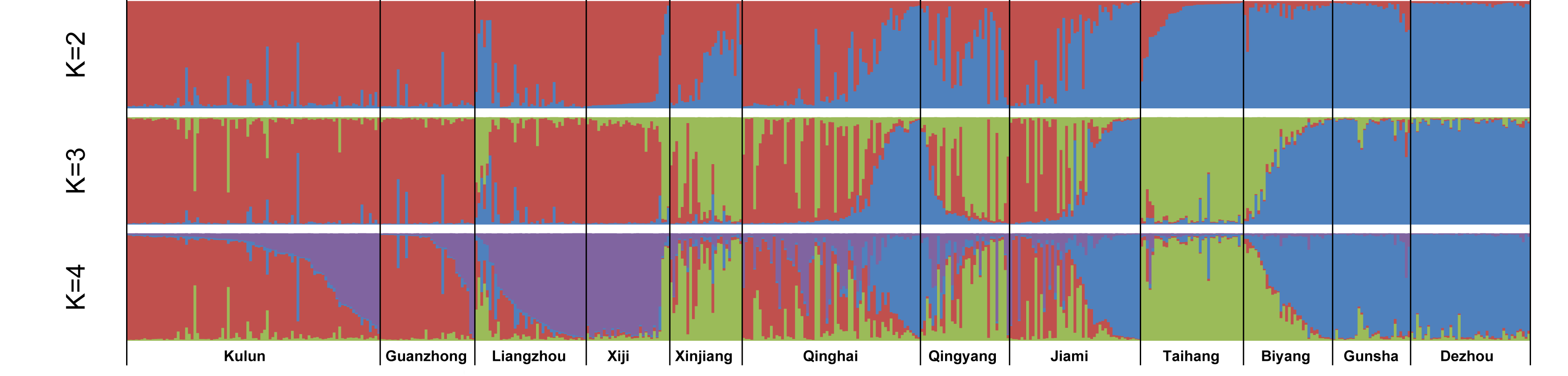

Supplement: The supplement related to this article is available online at: https://doi.org/10.5194/aab-62-181-2019-supplement. [file aab-62-181-supplement.zip › Supplement/Fig S1.tif]
